# Nitrous Oxide Abuse Complications in the Emergency Department: A Case Report

**DOI:** 10.3390/reports8030179

**Published:** 2025-09-16

**Authors:** Antonio Benjamin Lembo, Birgit Andrea Gartner, Matthieu Genoud

**Affiliations:** 1 Division of General Internal Medicine, Geneva University Hospitals, 1205 Geneva, Switzerland; 2 Division of Emergency Medicine, Department of Acute Care Medicine, Geneva University Hospitals, 1205 Geneva, Switzerland

**Keywords:** nitrous oxide, myelopathy, emergency medicine, B12 deficiency, neurological complications, addiction, abuse, social stigma, public health

## Abstract

**Case Presentation:** This report describes a case of acute severe myelopathy attributed to nitrous oxide abuse in a 30-year-old patient presenting with gait instability. **Background and Clinical Significance:** It depicts the challenges of early recognition in a primary care setting, and highlights through an individual patient-centered experience the profound social impacts of nitrous oxide abuse. **Discussion and Conclusions:** This case underscores the need for structured diagnostic approaches, interdisciplinary care, and further research to optimize treatment protocols and prevention strategies to warn nitrous oxide-related complications. Finally, this case quotes both key clinical and paraclinical screening indicators to facilitate case identification in emergency and primary care settings.

## 1. Introduction and Clinical Significance

Nitrous oxide (N_2_O) consumption for recreational purposes is a growing public health concern, particularly among young adults in Europe [1,2]. Although recreational use of N_2_O is widespread and severe neurological complications (such as subacute combined degeneration of the spinal cord) have been described, the trivialization of its use in social settings and the lack of consumer awareness would likely delay its recognition and management. In addition to these issues, emergency department (ED) physicians may encounter difficulties identifying N_2_O-related complications, presumably due to nonspecific symptoms and limited awareness, although the available evidence on this topic remains limited [1,3].

We report the case of a patient presenting with gait instability which was consecutive to an acute severe myelopathy caused by chronic N_2_O abuse.

Through this clinical vignette, we aim to highlight the diagnostic challenges encountered in the emergency setting and to explore the socio-environmental context of N_2_O use through the patient’s perspective. Furthermore, this case raises the interest of compiling some clinical and biological key indicators which could help case identification and prevention.

## 2. Case Presentation

A 30-year-old man known for attention deficit hyperactivity disorder and regular recreational substances use (cocaine, tobacco, and cannabis) was admitted to the ED for progressive walking impairment. His current history revealed increasing consumption of N_2_O, reaching up to 150 balloons a day. Progressive neurological symptoms appeared, characterized by painful dysesthesia, and weakness in the lower limbs leading to falls. During a general medical consultation, his condition had been firstly considered consistent with general fatigue and no further investigations were performed. Upon arrival in the ED, neurological examination revealed apallesthesia in both hallux with proximal increment and hypopallesthesia in the right upper limb. Muscle strength was preserved, reflexes were brisk and symmetrical, cranial nerves and higher functions were intact, without Lhermitte’s or pyramidal signs. However, the patient had extremely unstable gait. Initial laboratory tests revealed normal electrolytes and preserved renal, thyroid, and hepatic function, with no inflammatory markers. The complete blood count was within normal ranges, and D-dimer levels below 500 µg/L. Additional tests revealed normal folate levels but showed cyanocobalamin deficiency as evidenced by elevated methylmalonic acid (1.29 µmol/L; reference range < 0.28 µmol/L) and hyperhomocysteinemia (104 µmol/L; reference range < 15 µmol/L). Differential diagnoses included toxic combined cord sclerosis, sensory Guillain-Barré syndrome, Myelin Oligodendrocyte Glycoprotein Antibody-associated Disease (MOGAD), spinal cord ischemia and transverse myelitis.

In-hospital MRI revealed acute cervicothoracic centromedullary myelopathy while electroneuromyography (ENMG) showed signs of length-dependent axonal motor neuropathy consistent with acquired vitamin B_12_ deficiency (detailed results are provided in the Supplementary Materials). A diagnosis of acute toxic cervicothoracic centromedullary myelopathy secondary to heavy N_2_O abuse was established, as the patient refused lumbar puncture. The patient received high-dose intramuscular cyanocobalamin combined with physiotherapy and nutritional counseling.

Over the following month, walking gradually improved, though gait instability and upper limb fine motor deficits persisted. The patient remained adherent to treatment and no further diagnostic testing was deemed necessary.

## 3. Discussion

### 3.1. Diagnostic and Management Challenges in the ED

Nitrous oxide has long been used in the medical field for its analgesic and sedative properties. However, its recreational use has dramatically increased during the last years in many countries including Switzerland, particularly among young adults, leading to a rise in cases of intoxication. Since 2017, the introduction of recreationally targeted packaging has further sustained this trend, as highlighted by the European Monitoring Centre for Drugs and Drug Addiction [2]. Despite the increasing trend of N_2_O abuse and its well-documented effects, Swiss emergency physicians’ knowledge appears heterogenous and might trigger suboptimal care and missed diagnosis, as assumed in other European countries [3].

Among the numerous complications associated with N_2_O consumption, some are acute such as hypoxia due to rapid alveolar diffusion or the development of pneumothorax caused by accumulation of gas into closed cavities. Other complications may appear later, including hematological complications such as thromboembolic events due to acquired hyperhomocysteinemia, and megaloblastic anemia [4].

Our case focuses specifically on some of the neurological complications associated with N_2_O consumption. From a clinical standpoint centered on neurological consequences of N_2_O abuse, the central and peripheral nervous systems are primarily affected [5,6]. Neurological complications, primarily driven by acquired cyanocobalamin deficiency, are among the most common and functionally debilitating consequences. Symptoms are thought to express interferences of neurotransmission through pathway modulation involving glutamate, opioids, noradrenaline, and GABA explaining distortions of perception, analgesia, and dissociative states [7]. Less frequently, psychiatric manifestations such as hallucinations, dissociative states, and emotional instability may emerge, also supporting the abovementioned neurochemical mechanisms [8]. Regarding peripheral nervous system, patients typically present with sensory disturbances such as paresthesia and proprioceptive ataxia resulting from acute demyelination [9]. As exposure increases, some patients experience motor deficits, such as gait disturbances and lower limb weakness, which may evolve into myeloneuropathy mimicking subacute combined degeneration of the spinal cord [10,11,12]. This pattern of lesions is indicative of a functional cyanocobalamin deficiency resulting from the disruption of its metabolism by N_2_O.

The wide range of clinical presentations complicates early recognition, particularly in emergency settings with limited time and resources. Furthermore, evidence suggests that integration of cutting-edge diagnostic tools contributes to operational efficiency and improved quality of care [13]. Given these challenges, structured clinical reasoning remains essential. Identifying key neurological, biological, and behavioral clues as soon as possible can probably help guide appropriate investigations and prevent irreversible complications.

### 3.2. Socio-Environmental Insights

A structured follow-up interview was conducted with the patient six months after discharge, with the aim not only of exploring his experiences and social environment, but also of identifying the psychosocial factors contributing to escalating N_2_O use and gathering hints for prevention. strategies.

This testimony shows that access to N_2_O at the individual level remains surprisingly easy, largely facilitated by social media, in a context of silence surrounding its distribution channels and the normalization of its consumption, as previously highlighted by the EMCDDA [2,14]. The patient emphasized the particularly marked social and emotional repercussions of N_2_O abuse, reinforced by the prejudices associated with the use of addictive substances. These biases in clinical assessment can contribute to stigmatization in various social settings, there by reinforcing the minority stress model [15]. The patient reported a particularly traumatic episode: collapsing on the street, ignored by passers-by, which reinforced his feeling of isolation, already fueled by his perceived lack of interest from healthcare professionals. This loss of confidence led to disengagement from treatment, leading to continued substance use and worsening neurological symptoms until he was admitted to the ED.

Peer influence is one of the factors identified as impacting the outcomes of substance users [16]. On this subject, it is interesting to note that, the patient pointed out the inconsistency in social behavior: N_2_O is discouraged by his peers, but its continued use in a party setting reinforces his perception that it is not particularly dangerous.

Interestingly, the patient suggested using social media platforms such as Instagram or Snapchat to raise awareness and optimize the dissemination of information in the target population, similar to other applications developed fort tailored prevention campaigns [17]. These could address certain specific themes like cyanocobalamin supplementation aligning with EMCDDA recommendations [18], and most importantly preventive measures like limiting binge consumption to reduce the risk of hypoxia. It seems additionally crucial to emphasize the major risks involved when driving under nitrous oxide influence.

Moreover, a caring and non-judgmental approach is essential to limit the obstacles encountered by marginalized patients, who often have difficulty accessing care and are at increased risk of delayed or misdiagnosis. In this context, brief interventions carried out in emergency situations have proven effective, not only for alcohol-related disorders, but also for other substance use disorders. These short interventions represent a valuable opportunity to initiate follow-up care, prevent long-term complications and improve overall health [19].

### 3.3. Development of a Screening Table: The BALLON Questionnaire

Considering the diagnostic challenges in detecting complications of regular N_2_O exposure, a structured clinical approach appears essential, particularly in emergency and primary care settings. While numerous validated tools exist to detect problematic alcohol or substance use, to our knowledge none has yet been developed to specifically address N_2_O related risks and complications in acute care contexts [19].

Based on our patient’s case, we identified key variables for screening complications related to risky nitrous oxide use in the emergency department. These include the substance, the mode of consumption (commonly Balloons [18,20]) and the Amount (based on epidemiological data, critical threshold of >10 balloons/day may indicate binge use and increased neurotoxic risk [20,21]), the experience of Loss of control (as in DAST [19]), neurological and other somatic complications (L and O [10,11]), use of other substances and the irresistible urge to consume to relieve withdrawal [19]. Collectively, these items may guide early identification of high-risk users and facilitate timely intervention. (Table 1)

Of course, these points, if intended to form the basis of a clinical score or decision-support tool, would require several additional development steps. First, the objective should be clearly specified, whether focused on somatic complications, psycho-social risks or clinical guidance. Relevant variables as clinical, biological, radiological, and psycho-social, would need to be selected from observational data and weighted to create a practical score. The tool would then undergo internal and external validation to ensure diagnostic performance, robustness, and generalizability. Finally, implementation would require a simple format and training for healthcare professionals, enabling the integration of clinical, biological, and psycho-social dimensions in the assessment of nitrous oxide–related risks.

The BALLOON tool is exploratory and has not been validated to date. It is proposed as a structured clinical prompt derived from frontline experience, not as a diagnostic tool. Its potential utility must be confirmed through dedicated research, including prospective studies assessing diagnostic accuracy, feasibility, and user acceptance in real-life emergency settings.

**Table 1:** The BALLOON questionnaire.

**B**	**B**alloon use	Does the patient inhale nitrous oxide, such as through balloons?
**A**	**A**mount	Is the patient consuming a potentially risky amount of nitrous oxide, such as more than 10 balloons per session?
**L**	**L**oss of control	Does the patient encounter difficulties in reducing or stopping nitrous oxide use despite critics and clinical consequences?
**L**	**L**imb & neurological symptoms	In the recent medical history, is there sensory or motor disturbances, such as numbness or weakness in the extremities, and gait instability new psychiatric symptoms?
**O**	**O**ther manifestations	Does the clinician identify other signs of nitrous oxide toxicity, such as anemia, thrombosis, hypoxia, pneumothorax?
**O**	**O**ther substances	Does the patient use other drugs, which may exacerbate the effects of nitrous oxide, or indicate a potential addictive risk behavior?
**N**	**N**eed to use for relief	Is nitrous oxide consumed to relieve stress, anxiety, or physical discomfort?

One balloon refers to approximately 8 g of nitrous oxide, or about 4 L of gas under standard conditions (25 °C, 1 bar; density = 1.799 g/L).

## 4. Limitations

Our study has several limitations that should be mentioned. It is a single case report including individualized semiology and numerous data from an interview, the interpretation of which may be subjective. The practical screening table is a proposal which, as we have already mentioned, requires validation in future studies.

## 5. Conclusions

In conclusion, N_2_O consumption represents a rising public health concern that requires structured diagnostic protocols and a non-judgmental clinical approach, due to its potential neurological and socio-environmental consequences. Further validation and refinement of the screening table proposed here represent the next step in order to improve detection in ED settings.

## Data Availability

The original contributions presented in this study are included in the article. Further inquiries can be directed to the corresponding author.

## Supplementary Materials

The following supporting information can be downloaded at: https://www.mdpi.com/article/10.3390/reports8030179/s1.

## Abbreviations

The following abbreviations are used in this manuscript:

N_2_O	Nitrous oxide
ED	Emergency department
EMCDDA	European Monitoring Centre for Drugs and Drug Addiction

## References

[1] McCormick J.P., Sharpe S., Crowley K., Dudley A., O’lAoi R., Barry M., Owens L., Doherty C.P., Redmond J., Yeung S.-J. (2023). Nitrous oxide-induced myeloneuropathy: An emerging public health issue. Ir. J. Med. Sci..

[2] European Monitoring Centre for Drugs and Drug Addiction (2022). European Drug Report 2022: Trends and Developments.

[3] Riccò M., Ferraro P., Corrado S., Bottazzoli M., Marchesi F. (2023). Nitrous Oxide Inhalant Abuse: Preliminary Results from a Cross-Sectional Study on Knowledge, Attitudes, and Practices of Italian Physicians (2023). Medicina.

[4] Xiang Y., Li L., Ma X., Li S., Xue Y., Yan P., Chen M., Wu J. (2021). Recreational Nitrous Oxide Abuse: Prevalence, Neurotoxicity, and Treatment. Neurotox. Res..

[5] Sood R., Parent T.D.M. (2022). Peripheral polyneuropathy and acute psychosis from chronic nitrous oxide poisoning: A case report with literature review. Medicine.

[6] Thayabaran D., Burrage D. (2021). Nitrous oxide-induced neurotoxicity: A case report and literature review. Br. J. Clin. Pharmacol..

[7] Brunt T.M., Brink W.v.D., van Amsterdam J. (2022). Mechanisms Involved in the Neurotoxicity and Abuse Liability of Nitrous Oxide: A Narrative Review. Int. J. Mol. Sci..

[8] Paulus M.C., Wijnhoven A.M., Maessen G.C., Blankensteijn S.R., van der Heyden M.A.G. (2021). Does vitamin B12 deficiency explain psychiatric symptoms in recreational nitrous oxide users? A narrative review. Clin. Toxicol..

[9] Gernez E., Lee G.R., Niguet J.-P., Zerimech F., Bennis A., Grzych G. (2023). Nitrous Oxide Abuse: Clinical Outcomes, Pharmacology, Pharmacokinetics, Toxicity and Impact on Metabolism. Toxics.

[10] Pichon M., Majhadi L., Menn A.M. (2024). Neurological Manifestations Induced by Nitrous Oxide Abuse: A Case Series and Review of Literature. Neurologist.

[11] Marsden P., A Sharma A., Rotella J. (2022). Review article: Clinical manifestations and outcomes of chronic nitrous oxide misuse: A systematic review. Emerg. Med. Australas..

[12] Winstock A.R., A Ferris J. (2020). Nitrous oxide causes peripheral neuropathy in a dose dependent manner among recreational users. J. Psychopharmacol..

[13] Mostafa R., El-Atawi K. (2024). Strategies to Measure and Improve Emergency Department Performance: A Review. Cureus.

[14] Allan J., Cameron J., Bruno J. (2022). A Systematic Review of Recreational Nitrous Oxide Use: Implications for Policy, Service Delivery and Individuals. Int. J. Environ. Res. Public Health.

[15] Dahl R.A., Vakkalanka J.P., Harland K.K., Radke J. (2022). Investigating Healthcare Provider Bias Toward Patients Who Use Drugs Using a Survey-Based Implicit Association Test: Pilot Study. J. Addict. Med..

[16] van de Ven K., Stainthorpe S., Davies A., Cash R., Ross P., Lee N. (2025). A scoping review of key domains for youth outcome measurement in alcohol and other drug treatment. Int. J. Drug Policy.

[17] Schwinn T.M., Fang L., Hopkins J., Pacheco A.R. (2021). Longitudinal Outcomes of a Smartphone Application to Prevent Drug Use Among Hispanic Youth. J. Stud. Alcohol Drugs.

[18] van Aerts L., de Morais J., Evans-Brown M., Jorge R., Gallegos A., Christie R., Néfau T., Planchuelo G., Sedefov R., European Monitoring Centre for Drugs and Drug Addiction (2022). Recreational Use of Nitrous Oxide: A Growing Concern for Europe. https://www.euda.europa.eu/publications/rapid-communication/recreational-use-nitrous-oxide-growing-concern-europe_en.

[19] Pautrat M., Lebeau J.P., Laporte C. (2022). Identifying available addictive disorder screening tests validated in primary care: A systematic review. Addict. Behav..

[20] van Amsterdam J., Nabben T., van den Brink W. (2015). Recreational nitrous oxide use: Prevalence and risks. Regul. Toxicol. Pharmacol..

[21] Kaar S.J., Ferris J., Waldron J., Devaney M., Ramsey J., Winstock A.R. (2016). Up: The rise of nitrous oxide abuse. An international survey of contemporary nitrous oxide use. J. Psychopharmacol..

