# Supplementary material for: Nitrous Oxide Abuse Complications in the Emergency Department: A Case Report
Source: Reports (MDPI). 2025 Sep 16;8(3):179. doi: 10.3390/reports8030179 (PMC12452426; doi:10.3390/reports8030179)
Supplement: Supplementary file 1 [file reports-08-00179-s001.zip › reports-3772349-supplementary.pdf]

Supplementary Material
Appendix 1 – Patient Testimony

Introduction:
Case reports rarely include direct interviews with the patients concerned. In this appendix, we share selected excerpts from an interview conducted with the patient described in our report. His personal account provides additional perspective on the patterns of use, perceived effects, and emotional experiences associated with nitrous oxide (N_2O), complementing the clinical description.

Access to Nitrous Oxide and Purchase Conditions
Q1: How did you obtain nitrous oxide? Was it difficult? Were vendors concerned about the quantities you were buying? What were the prices like, and did you pay by card, cash, or online? What was your overall budget?

A1: I used to buy industrial whipped cream chargers quite easily, without vendors questioning the quantities I purchased. In Switzerland, I typically paid between 20 and 30 CHF per canister, and around 15 euros in France, with offers like 50 euros for three canisters. The sellers knew what I was using them for but still provided them without asking questions. I found that some brands were less pleasant to use — they caused significant numbness without the euphoric effect I was seeking (specifically a blue and white bottle). In my opinion, the best brand was called La Crème. Later, I started getting deliveries from private individuals. I also knew that through platforms like Snapchat or Telegram, prices in Geneva could go up to 50 or even 80 CHF per canister. These channels were essentially how everything was being distributed.

First Exposure to Nitrous Oxide
Q2: How did you learn how to use nitrous oxide?
A2: I was first introduced to nitrous oxide by some friends during the winter of 2022. I hyperventilated while inhaling the gas, which gave me an intense rush. I felt incredibly free, as if I were riding a horse.

Desired Effects and Evolution with Repeated Use
Q3: What effects were you seeking, and how did these effects change with repeated use?
A3: The main effect I was looking for was to calm myself down, especially at the end of the night, while listening to music and using nitrous oxide balloons. Sometimes, I would combine it with cocaine to enhance the effect. All tactile sensations were heightened, and I also had sexual experiences under its influence. Each time I used it, I felt like I was inside a bubble, isolated from the rest of the world. With repeated use, this bubble-like feeling became the refuge I was seeking at the end of the day — although I could still go without it if I wanted to.

Escalation of Use and Impact on Daily Life

Q4: How did your use of nitrous oxide increase over time?
A4: At first, three of us would share a single canister over the course of an hour. Gradually, I began using one canister by myself, and eventually, I would consume up to three canisters in a row. My consumption really escalated under stress. One event particularly stuck with me: I

had started feeling weakness in my legs, and one day I collapsed in the street. People around me simply continued on with their day, stepping over me without paying any attention. After that experience, I withdrew even more into myself.

Methods of Nitrous Oxide Use

Q5: How did you consume nitrous oxide? Were there any specific techniques you used?

A5: I used nitrous oxide by filling a balloon and breathing the same gas back and forth for about 3 to 5 minutes, creating a kind of asphyxiation effect that I referred to as "by suffocation." This method came to me naturally, and I consistently used the same technique to intensify the desired effect.

Awareness of Risks Before Starting

Q6: Were you aware of the risks associated with nitrous oxide before you started using it?

A6: Before I began using nitrous oxide, I wasn't really aware of the associated risks. I remember that the gas would come out cold and it would hurt, so I later changed my technique. But I had no idea about the more serious dangers. Even when people tried to warn me, it was pointless — it was like I didn't want to know or hear about it.

Precautions and Advice in Hindsight

Q7: Are there any precautions or advice you learned, or things you wish you had known before using nitrous oxide?

A7: If I had known about the importance of vitamin B12 and its connection to nitrous oxide use, I would have started taking supplements right away. At the beginning, I had no information about these risks. Toward the end, when I became aware of the need, I tried to get supplements, but without success. If I could go back, I would take every possible vitamin to avoid what happened.

Early Warning Signs Before Serious Symptoms

Q8: Were there any early warning signs that you might have overlooked before more serious symptoms appeared?

A8: Before the more serious symptoms set in, I felt a cold sensation in my feet that gradually moved up through my body. It persisted even outside of nitrous oxide use. I mentioned it to a doctor who didn't know me or my nitrous oxide use. He reassured me by diagnosing a streptococcal throat infection and prescribed antibiotics. He told me that feeling cold was normal. I wasn't really convinced, but he insisted there was nothing more to worry about. Despite my insistence, he did nothing further, and I lost trust in him. Later, I also had several brief episodes of loss of consciousness outside of any consumption, which I also ignored.

Perception of Nitrous Oxide Use Over Time

Q9: How did your perception of nitrous oxide evolve as your use continued?

A9: I never felt addicted to nitrous oxide — I had no cravings or real need to consume. It was mostly about the gesture. When I saw the canister, it made me want to use it because it was easy and accessible.

Impact on Social, Emotional, and Professional Life

Q10: How did nitrous oxide use impact your social life, emotional well-being, and work?
A10: It affected my ability to do my delivery job, as the symptoms forced me to take reckless risks, especially using an electric bike for transportation. Falls became frequent, and toward the end, I stopped going out altogether, which isolated me socially and worsened my emotional struggles.

Feelings of Guilt and Attempts to Cut Down (CAGE Questionnaire)
Q11: Did you ever feel shame or guilt about your use? Did you try to stop or cut down? Did you use it first thing in the morning? Has anyone ever commented on your use?
A11: No, I felt much more shame about cocaine. Nitrous oxide was completely normalized: nightclubs even sold it, fully aware of how it was being used.
Yes, I had tried to stop. If it wasn't available, it wasn't a problem. But when I was alone at home and bored, I would easily start using it again, or if friends came over to talk about their problems, I would use it to escape.
I did use it in the morning, right after waking up — especially if the balloon was already attached to the canister. That was my combo: cigarette and balloon.
Yes, many people told me to stop. Their advice was more about concern for my health, not about my behavior, which wasn't noticeably affected. But they weren't very credible, because after telling me to stop, they would still do balloons with me and say it wasn't so bad.

Medical Help-Seeking Without Police Intervention

Q12: Without the intervention of the police, would you have sought medical help? If so, when and with whom?
A12: No, I wouldn't have sought help without the police intervention. I had already seen doctors before, but I felt like they ignored my symptoms and didn't take my pain seriously. I had the impression they didn't really care about my situation. No one seemed to grasp how severe my pain was, even when I explained how the soles of my feet felt like they were being stabbed by needles, even when wearing socks or shoes.

Shifting from Other Substances to Nitrous Oxide
Q13: Why did you shift from using other substances to nitrous oxide?
A13: Cocaine was my main drug of choice, but after converting to Islam, I had to stop all forms of drug use. However, I didn't see nitrous oxide balloons as a real drug, which allowed me to keep using them without feeling like I was violating my beliefs.

Sources of Support and Information
Q14: What support resources (community-based, online, or healthcare-related) have you used or are you aware of?
A14: To find support, I started by searching on Google for information about the symptoms I was experiencing, such as the numbness related to balloon use. That's how I discovered the link with vitamin B12. I also had a friend who told me she had overcome similar symptoms by taking vitamin B12 supplements, which encouraged me to try the same approach to manage my own symptoms. However, the pharmacy refused to provide me with the right quantities. They said I needed a prescription, but I didn't have one and no longer trusted doctors.

Prevention Strategies for Nitrous Oxide Intoxication

Q15: What measures could be put in place to prevent similar cases of nitrous oxide intoxication?

A15: To prevent nitrous oxide intoxications, platforms like Instagram and Snapchat could be used to spread information about the risks and prevention strategies. For example, funding targeted ads on Instagram to raise awareness about the importance of vitamin B12 and making this information widely accessible could help educate the public proactively about the dangers and encourage safer consumption behaviors.

Conclusion:

This testimony offers a complementary view to the clinical findings, highlighting the personal and practical aspects of nitrous oxide use that may otherwise remain invisible in standard case reports.

Appendix 2

Images of MRI :

Central medullary hypersignal beginning at C2 level (image 1) and appearing to spare the periphery. From D2 to D10 level, the hypersignal becomes more prominent in the anterior portion of the spinal cord (image 2).

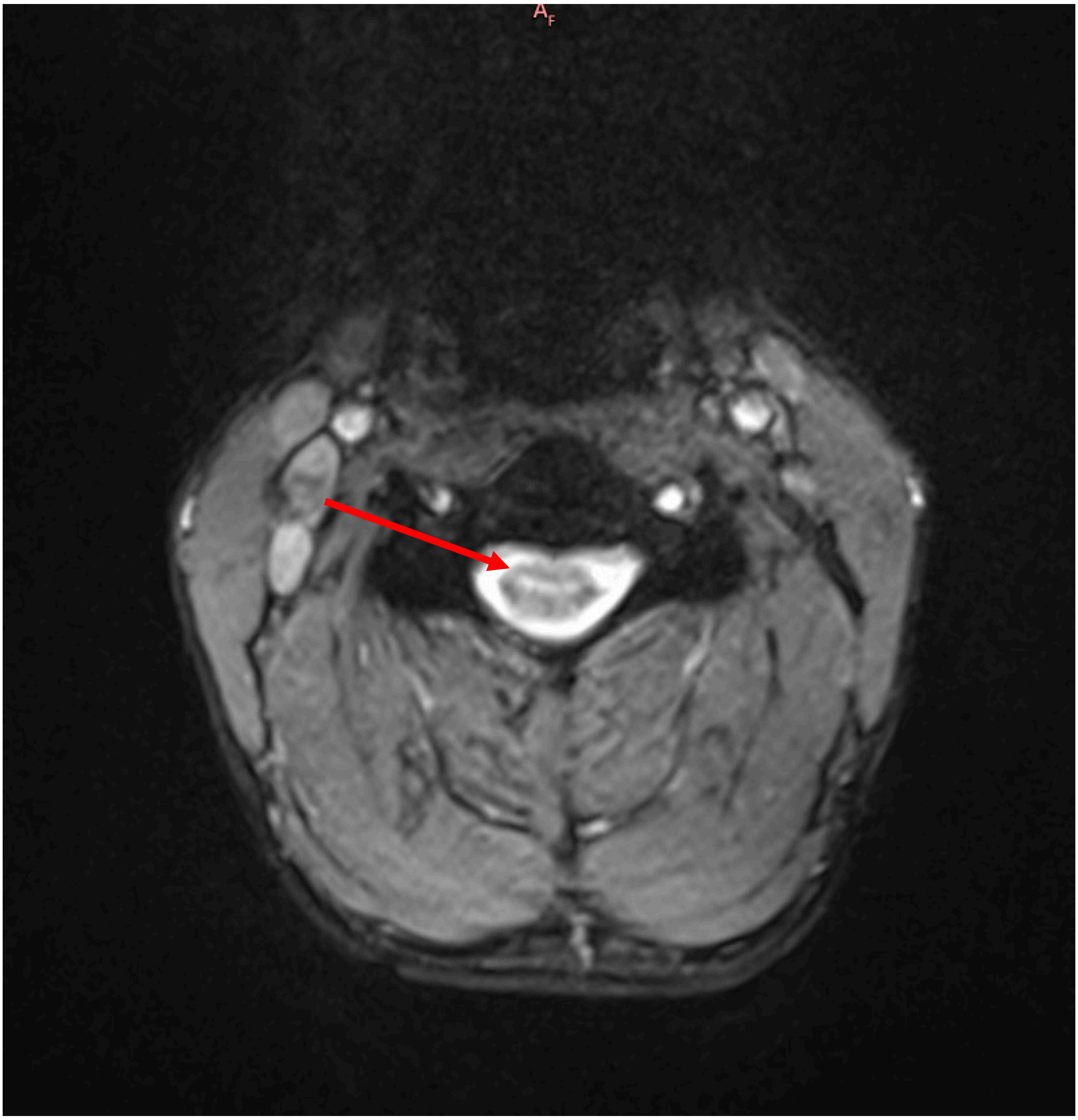

Image 1

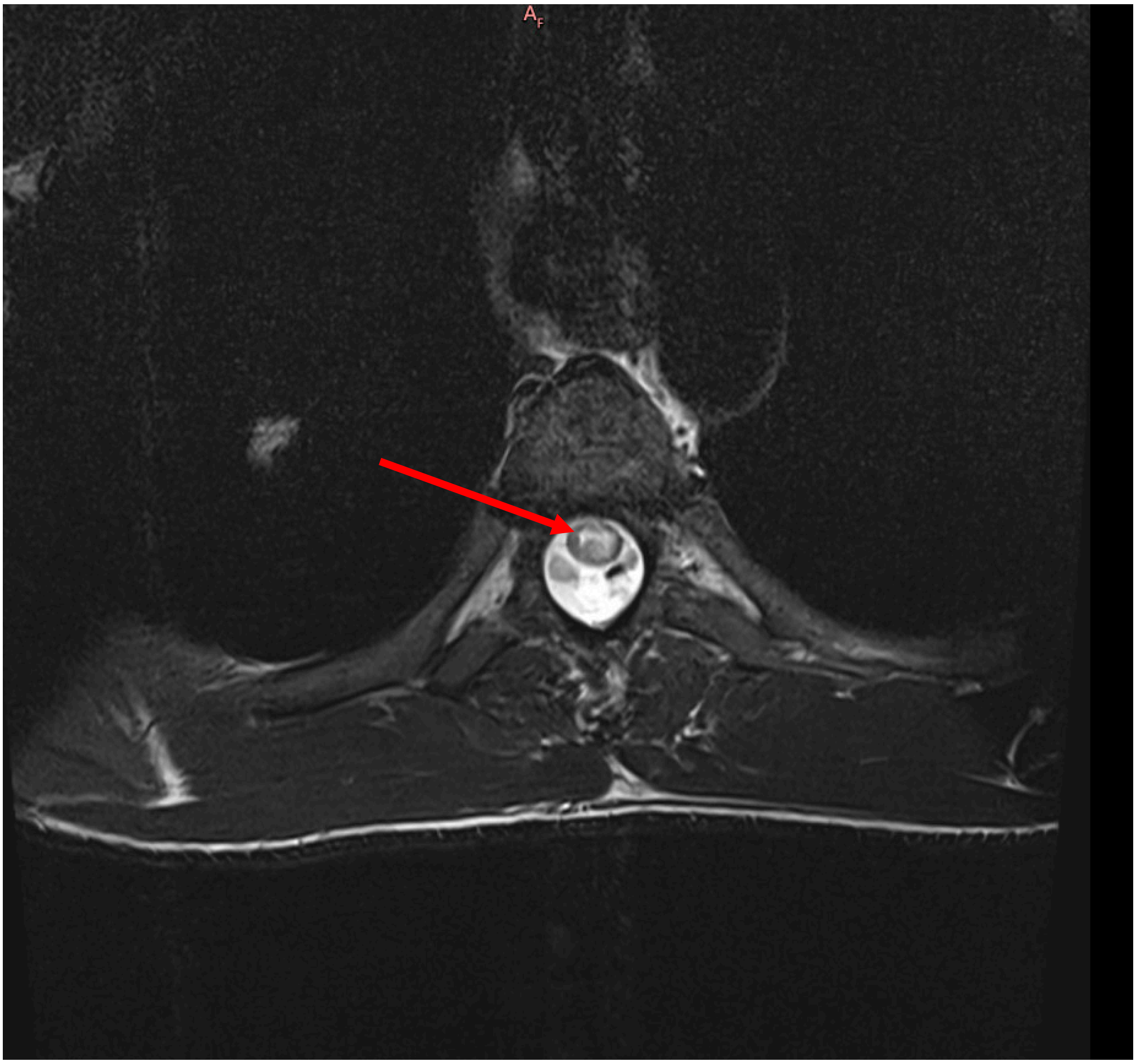

Image 2

Appendix 3

Results of ENMG

Signs of length-dependent motor axonal neuropathy consistent with a complication of nitrous oxide poisoning. The results are presented as they appear in the official report validated and signed by neurologists.

Sensitive

Nerf / Sites	Recueil	Lat1 ms	Dur. ms	d Lat. ms	Amp. µV	Dist. mm	CV m/s	Ralent. %
**G Radial**								
Av bras	Tabatière	1.8	1.4	1.8	38	90	51	
**G N. suralis, Superficial peroneal**								
N. suralis 1/3 inf. mollet	mall. ext.	1.9	1.6	1.9	22	70	37	
**D N. suralis, Superficial peroneal**								
N. suralis 1/3 inf. mollet	mall. ext.	2.2	1.5	2.2	17	90	41	

D N. suralis, Superficial peroneal: Spe

Motrice

Nerf / Sites	Lat1 ms	Dur ms	Amp mV	Surf. mVms	Dist mm	CV m/s	rAmpP %	rArea-%
**G Ulnaire, Médian - hypothénar**								
Ulnaire Poignet	2.8	6.2	9.7	33.8	65			
Ulnaire Coude sous	7.2	6.1	9.7	32.0	250	56	99.9	92.3
Ulnaire Coude sus	9.4	6.1	8.6	28.9	120	54	88.5	90.9
**D N. tibialis - abd.hallucis**								
Mal int	6.5	7.2	6.7	25.6	95			
Creux popl	15.8	7.8	5.0	24.6	400	43	74.6	88.1
**G Peroneal - EDB**								
Cou de pied	5.9	7.3	1.9	5.7	65			
Col dev.	13.5	8.8	1.6	7.0	300	39	82.8	91.6
Col derr.	16.0	8.7	1.6	7.4	110	45	102.9	105.0
**D Peroneal - EDB**								
Cou de pied	6.8	5.3	1.1	1.7				

Ondes F

Nerf	M Amp pp max mV	M-Lat ms	F-Lat ms	F-M Lat ms
**G Ulnaire**	7.92	2.8	30.4	27.6
**D N. tibialis**	4.50	6.3	53.9	47.7
**G Peroneal - tibialis ant.**	1.55	6.9	50.6	43.6
**D Peroneal - tibialis ant.**	0.94	5.0	47.1	42.1

EMG à l'aiguille

**EMG**										
	**Activité d'insertion**	**Activité spontanée**			**Contraction volontaire**			**PUM**		
**Muscle**	**Insertion**	**Fib/PP**	**Fasc.**	**autre**	**qualité**	**recrutement spatial**	**recrutement temporel**	**Amplitude**	**durée**	**forme**
**G. Tibialis anterior**	normale	0	+	0	Max.	Tinf.	Normal	normale	normale	normale

ABRÉVIATIONS

*	: valeur hors norme
//	: élément non examiné
DDi	: décharge double indirecte
DHF	: décharge de haute fréquence
Fasc.	: potentiel de fasciculation
Fib.	: potentiel de fibrillation
FR	: fréquence rapide
Myot.	: décharge myotonique
NR	: no response
PGPs	: positive giant potentials
Pp	: potentiel positif
PUM	: potentiel d'unité motrice
Synchr.	: tracé synchronisé
TI	: tracé intermédiaire
Tinf.	: tracé d'interférence
